# Effects of Different Calorie Restriction Protocols on Oxidative Stress Parameters in a Transgenic Mouse Model of Breast Cancer

**DOI:** 10.7759/cureus.27895

**Published:** 2022-08-11

**Authors:** Munevver B Cicekdal, Pinar B Thomas, Bilge Guvenc Tuna, Mohammad Charehsaz, Ahmet Aydin, Bayram Yilmaz, Margot P Cleary, Soner Dogan

**Affiliations:** 1 Department of Medical Genetics, Ghent University, Ghent, BEL; 2 Department of Medical Biology and Genetics, Maltepe University, Istanbul, TUR; 3 Department of Biophysics, School of Medicine, Yeditepe University, Istanbul, TUR; 4 Department of Pharmaceutical Toxicology, Yeditepe University, Istanbul, TUR; 5 Department of Physiology, Yeditepe University, Istanbul, TUR; 6 Department of Nutrition and Metabolism, Hormel Institute Medical Research Center, Austin, USA; 7 Department of Medical Biology, School of Medicine, Yeditepe University, Istanbul, TUR

**Keywords:** ageing, mouse model, breast cancer, oxidative stress, intermittent calorie restriction

## Abstract

Aging and diseases related to aging, such as cancer, have been linked to oxidative stress. On the other hand, calorie restriction (CR) is one of the most effective interventions to slow down aging and prevent a variety of diseases such as cancer in preclinical models. CR has also been reported to modify oxidative stress. The aim of this study was to investigate the effects of different CR protocols and aging on oxidative stress parameters in the MMTV-TGF-α breast cancer mouse model in a cross-sectional study. Female mice were randomly enrolled in three groups: ad libitum (AL), chronic calorie restriction (CCR, 15% CR) or intermittent calorie restriction (ICR, three weeks AL followed by one week 60% CR in cyclic periods) starting at the age of 10 weeks until 81/82 weeks of age. Liver samples were analyzed for malondialdehyde (MDA), catalase (CAT), superoxide dismutase (SOD), glutathione (GSH), and glutathione peroxidase (GSH-Px) levels. At week 49/50, the GSH level increased significantly in the CCR group compared to the AL and ICR-R groups which had higher mammary tumor (MT) incidence rates. Additionally, liver MDA levels in ICR groups were significantly increased, while aging led to decreased CAT and SOD activities in all CR groups. The application of different CR protocols did not have any significant effect on MDA, CAT, and SOD parameters in the liver at week 81/82. These results suggest that although GSH may interfere with MT development at the systemic level, many of the oxidative stress parameters may have more local effects on tumor development than the systemic effects.

## Introduction

Calorie restriction (CR) is a dietary regime that is based on reducing calorie intake without causing malnutrition. Effects of CR on aging and age-associated disorders have been investigated in a number of species including nematodes, fish, hamsters, mice, rats, and monkeys [1]. These studies suggested CR as one of the most effective interventions that improve both health and life span as it robustly slows down aging processes and prevents age-associated disorders including cardiovascular diseases, diabetes, neurodegeneration, and cancer [2].

Two main types of CR protocols, chronic calorie restriction (CCR) and intermittent calorie restriction (ICR), that are commonly applied result in a 20-40% reduction in overall calorie intake [3]. Meta-analysis studies have revealed that both types of CR interventions exhibit preventive anticancer effects as evidenced by significantly reduced tumor incidence rate in either CCR or ICR groups compared to AL group in animal models [4]. Although the molecular mechanisms of the anticancer effects of CR have been suggested to involve alterations in the expression and signaling pathways of a number of proteins including IGF-1, adipokines, mTOR, RAS/MAPK, and PI3K/Akt, the exact molecular mechanism(s) of positive effects of CR on cancer prevention is/are still not fully elucidated [5,6].

Oxidative stress is a state where there is an imbalance between the generation and detoxification of reactive oxygen species (ROS) in favor of the former. ROS are natural products of aerobic metabolism and include both free radicals and non-radical species, which have the ability to generate free radicals [7]. At low to moderate concentrations, ROS play beneficial roles in various physiological processes including the immune defense system and redox regulation of transcription factors. However, ROS levels higher than the physiological concentration cause oxidative damage to important cellular macromolecules including proteins, lipids, and DNA [8]. ROS levels are balanced at low to moderate concentrations by the antioxidant defense mechanisms under physiological conditions. However, cigarette smoking, toxicants, obesity, a high-fat diet, and/or aging may lead to the disruption of this balance [9,10]. The severity of the consequences of this imbalance depends on a variety of factors, including the level and the location of ROS production, the efficacy of antioxidant systems, and the cellular targets of ROS [11]. Aging and age-related diseases such as cancer, neurodegeneration, cardiovascular diseases, and diabetes have been attributed to oxidative stress [12-14]. Therefore, it is crucial to modulate intracellular ROS levels by the antioxidant defense mechanisms for the maintenance of redox homeostasis. In this manner, CR has been shown to have significant roles in oxidative stress balance and may play protective roles against cancer, neurodegeneration, and cardiovascular disease [15]. However, whether ICR has any impact on oxidative stress levels in the preventive effects of CR in cancer development is still not clear. Therefore, the present study aims to measure and compare the systemic effects of different CR protocols on oxidative stress parameters in the liver tissue of an aging transgenic breast cancer mouse model, MMTV-TGF-α mice in a cross-sectional way. We report, for the first time, the effects of long-term application (up to 72 weeks) of different calorie restriction types (CCR and ICR) on oxidative damage (malondialdehyde (MDA)) and antioxidant markers (superoxide dismutase (SOD), catalase (CAT), glutathione (GSH), and glutathione peroxidase (GSH-Px)) in the liver tissue of MMTV-TGF-α mice. We also examine the effects of aging on oxidative stress parameters in mice subjected to different CR protocols.

## Materials and methods

Animals and experimental design

MMTV-TGF-α (C57BL/6) positive female mice were used in this study. Four MMTV-TGF-α (C57BL/6) positive male mice, kindly provided by Dr. Margot Cleary, Hormel Institute Medical Research Center, University of Minnesota, were used to establish a breeding colony at Yeditepe University Medical School Experimental Research Center (YUDETAM). Mice used in the present study are good models for post-menopausal breast cancer [16]. The mice were prone to develop mammary tumor (MT) since they were all genetically confirmed to overexpress the MMTV-TGF-α gene, which leads to overgrowth of mammary epithelial cells. Mice in the present study did not develop any MT during the course of the study; however, if the mice were kept alive for a longer period of time, most, if not all, of them would have developed MT. Thus, tumor development factors did not interfere with the CR and aging factors on oxidative stress. Since wild-type mice were used in previous studies investigating the effects of CR on oxidative stress and yet the ICR effects on oxidative stress have not been reported, the present study will potentially have a significant contribution to the literature. All mice had free access to tap water. The health status of the mice was checked by an expert veterinarian regularly, at least once a week. Female mice were randomly assigned into three groups at 10 weeks of mouse age: ad libitum (AL), chronic CR (CCR), or intermittent CR (ICR) [12]. Mice diets (Altromin TPF1414) were purchased from Kobay AS (Ankara, Turkey). AL group of mice had free access to food throughout the study, while mice in the CCR group were provided 85% of the daily food consumed by the age-matched AL group. In other words, the calorie intake of the CCR group was reduced by 15% relative to the AL group. On the other hand, mice in the ICR group were given 40% of the age-matched AL group of mice for one week and then they were fed AL for the following three weeks in a cyclic manner until the designated sacrifice time points. After overnight fasting, mice were sacrificed and liver samples were collected at designated ages: 10 (baseline), 17, 18; 49, 50; and 81, 82 weeks of age. Mice in the ICR group were divided into two subgroups; half of the mice in the ICR group were sacrificed at the end of the three-week AL feeding period (weeks 17, 49, and 81), and these groups were called ICR-refeeding (ICR-RF). The other half of the ICR group were sacrificed and the liver samples were collected at the end of the one-week CR period (weeks 18, 50, and 82), and these groups were called ICR-restricted (ICR-R). Since data from two consecutive weeks (17 and 18; 49 and 50; 81 and 82 weeks) were similar between the AL and CCR groups, the results for these groups at the designated time points were combined. All experimental procedures in animals were performed under the guidelines and with the approval of the Yeditepe University Animal Care and Use Committee (Approval # 371, 01.31.2014).

Preparation of liver homogenates

Liver tissue samples were collected and snap frozen in liquid nitrogen at sacrifice. At the time of preparation, liver samples were sliced and 100 mg of each liver sample was placed into 1.5 ml Eppendorf tubes. One measure of 0.05 mm zirconium magnetic beads was added to each sample. Following that, 9 volumes of 1.15% potassium chloride (KCl) solution were added to the liver samples, which were homogenized by Bullet Blender homogenizer. After homogenization, the samples were centrifuged at 1000 *g* for 10 minutes at +4° C and the supernatants were removed. Supernatants were aliquoted and stored at -80°C until analysis. The Lowery method was used to determine the protein content in the supernatants.

Measurement of oxidative stress parameters

Lipid Peroxidation

MDA levels in the liver were measured to determine lipid peroxidation using a method by Jamall and Smith [17], which is based on the reaction between MDA and thiobarbituric acid (TBA; Sigma Aldrich, St Louis, MO; T5500), resulting in a measurable pink color. Briefly, 0.2 ml of liver homogenate, 0.2 ml 8.1% sodium dodecyl sulfate solution, 1.5 ml 0.8% TBA, and 0.6 ml of water were mixed and incubated at 95°C for 1 hour. Following the incubation, 2 ml 10% trichloroacetic acid (Sigma Aldrich, 27242) solution was added to 2 ml of the mixture and centrifuged at 1000 *g* for 10 minutes. The absorbance of the supernatant was determined at 532 nm using a spectrophotometer (Thermo Scientific Evolution 300, Waltham, MA). MDA level in each sample was calculated using the standard curve.

Measurement of antioxidative defense parameters

CAT Activity

Antioxidant capacity was examined by determining the CAT activity according to Aebi’s method, which is based on measuring the hydrogen peroxide scavenging activity of CAT in the samples. Briefly, 1 ml of 10 mM hydrogen peroxide (Sigma Aldrich, 18304) was added to 2 ml of samples, and CAT activity was monitored spectrophotometrically at 240 nm every 30 seconds 3 times. CAT activity level was assessed in each sample using the standard curve.

SOD Activity

SOD activity was measured according to a previously described method by Sun et al. [18]. This method relies on the reaction between superoxide and iodonitrotetrazolium (INT), which results in a pink color, and measuring the decrease in the intensity of the pink color spectrophotometrically at 505 nm over a three-minute period. Briefly, a substrate solution containing 0.05 mmol/l xanthine sodium (Sigma Aldrich, X2502), 0.025 mmol/l INT (Sigma Aldrich, I10406), 50 mmol/l CAPS (Sigma Aldrich, C2632), and 0.94 mmol/l ethylenediaminetetraacetic acid (EDTA; Sigma Aldrich, E5134) were added to 25 µl of the sample. Then, 125 µl of xanthine oxidase (80 U/l) was added to the mixture. The color change was analyzed every 30 seconds for 3 minutes by a spectrophotometer and the SOD activity level was examined in each sample using the standard curve. 

GSH Levels

GSH levels were determined using Ellman’s method, which is based on the reaction between Ellman’s solution and sulfhydryl groups resulting in a colorimetrically measurable yellow color. In brief, a precipitant solution composed of metaphosphoric acid, disodium EDTA, and sodium chloride (NaCl, Sigma Aldrich, 13423) was added to a 200 µl sample and centrifuged for 30 minutes at 3000 rpm. After that, 100 µl of supernatant was mixed with disodium phosphate (Na_2_HPO_4_) and 0.02% 5,5′-dithiobis (2-nitrobenzoic acid) (DTNB). The change in color was measured by a spectrophotometer at 412 nm. The following formula was used to calculate the level of GSH: GSH (µmol/ml) = OD × 37.23, where OD is the optical density.

GSH-Px Levels

GSH-Px levels were determined as described previously. Briefly, samples were diluted with dH_2_O in order to measure GSH-Px activity in 1:5 dilutions for erythrocytes and in a 1:17 ratio for tissue homogenates. Then, 10 μl of each sample was mixed with 990 μl of the reaction mixture that contains 1 mmol/l of EDTA disodium dihydrate, 2 mmol/l of reduced GSH, 0.2 mmol/l of NADPH, 4 mmol/l of sodium azide, and 1000 U of glutathione reductase in 50 mmol/l tris buffer. The mixture was incubated for 5 minutes at room temperature and 10 μl of tert-butyl hydroperoxide (1:1000 dilution) was added. Quartz cuvette was immediately placed in a spectrophotometer and measurements were taken at 340 nm every 30 minutes for 3 minutes. Following the measurements, GSH-Px activity was calculated according to the following formula; Activity U/l = (∆Abs/0.00622) × 100. 

Statistical analysis

Statistical analyses were performed using GraphPad Prism version 5 (GraphPad Software, Inc., San Diego, CA). Data are presented as mean ± standard error of the mean (SEM). One-way analysis of variance (ANOVA) followed by the Bonferroni multiple comparison test was used to perform comparisons among different groups. The correlation between two parameters was analyzed using Pearson's correlation test and linear regression analysis separately. “n” represents the number of samples collected from different animals. Differences are considered statistically significant when p <0.05.

## Results

MDA levels in liver

**Figure 1:**
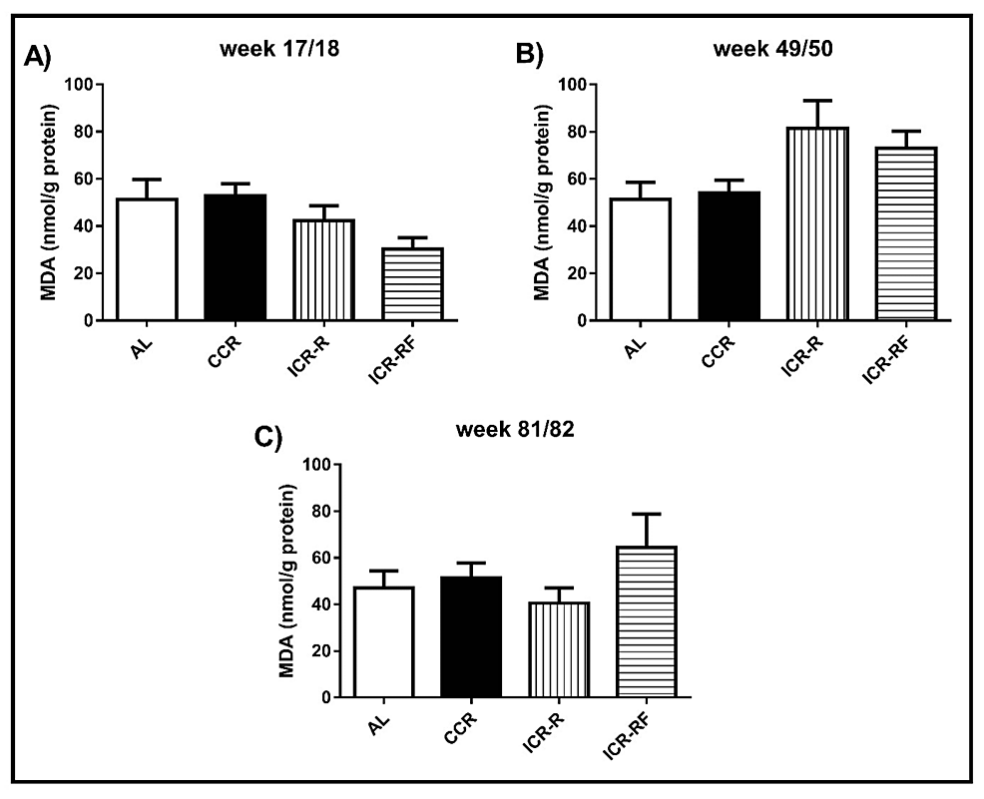
Effects of different types of calorie restriction on MDA level in liver Effects of different CR types on MDA level at week 17/18 (n = 9-18, Panel A), at week 49/50 (n = 11-16, Panel B) and at week 81/82 (n = 5-12, Panel C). Values represent the mean ± SEM. One-way ANOVA followed by the Bonferroni multiple comparison test was used to perform comparisons among different groups. * represents significant difference (p < 0.05). AL = ad libitum; CCR = chronic calorie restriction; ICR-R = intermittent calorie restriction-restricted; ICR-RF = intermittent calorie restriction-refeed; MDA = malondialdeyde; SEM = standard error of the mean; ANOVA = analysis of variance.

Liver MDA levels in all dietary groups (AL, CCR, ICR-R, and ICR-RF) at 17/18 weeks, 49/50 weeks, and 81/82 weeks of age are shown in Figures 1A, 1B, 1C, respectively. No significant differences in liver MDA levels among dietary groups were detected. Notably, although not statistically significant, ICR-R and ICR-RF groups had about 25% to 35% higher MDA levels compared to either AL or CCR group at week 49/50 (p > 0.05, Figure 1B).

CAT activity levels in liver

**Figure 2:**
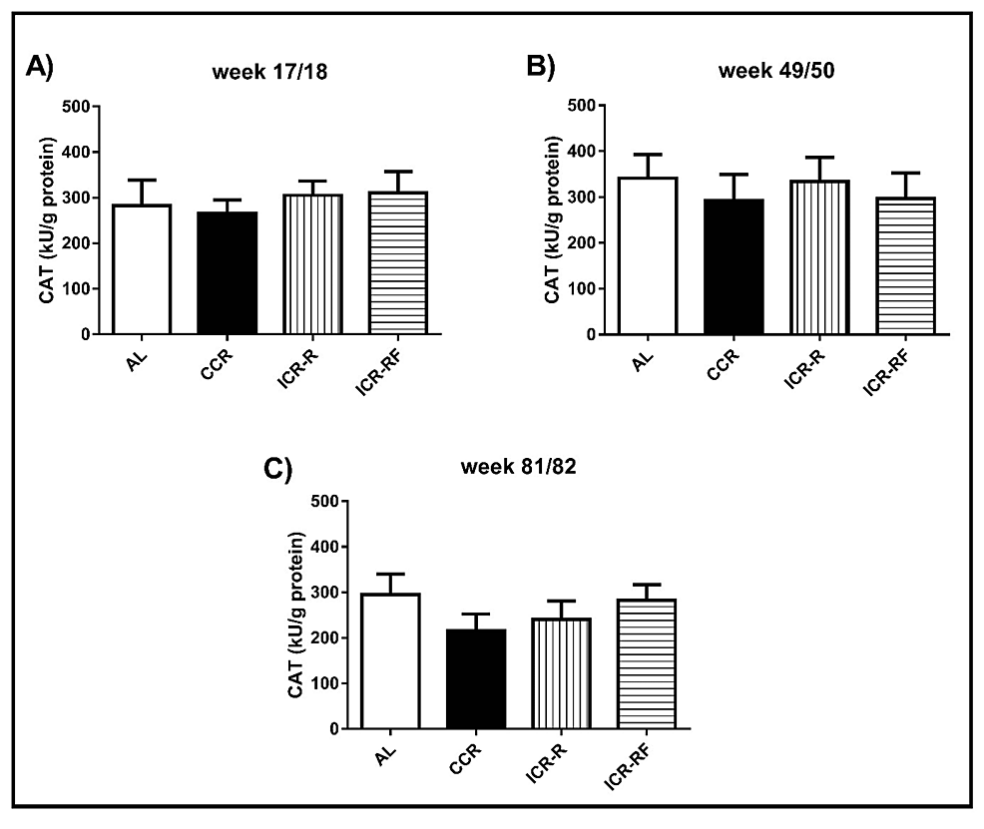
Effects of different types of calorie restriction on CAT activity in liver Effects of different CR types on CAT activity at week 17/18 (n = 13-17, Panel A), at week 49/50 (n = 9-16, Panel B), and at week 81/82 (n = 5-12, Panel C). Values represent the mean ± SEM. One-way ANOVA followed by the Bonferroni multiple comparison test was used to perform comparisons among different groups. * represents significant difference (p < 0.05). AL = ad libitum; CCR = chronic calorie restriction; ICR-R = intermittent calorie restriction-restricted; ICR-RF = intermittent calorie restriction-refeed; CAT = catalase; SEM = standard error of the mean; ANOVA = analysis of variance.

No significant differences in liver CAT activity were found among the dietary groups at any time point (p > 0.05, Figure 2).

SOD activity levels in liver

**Figure 3:**
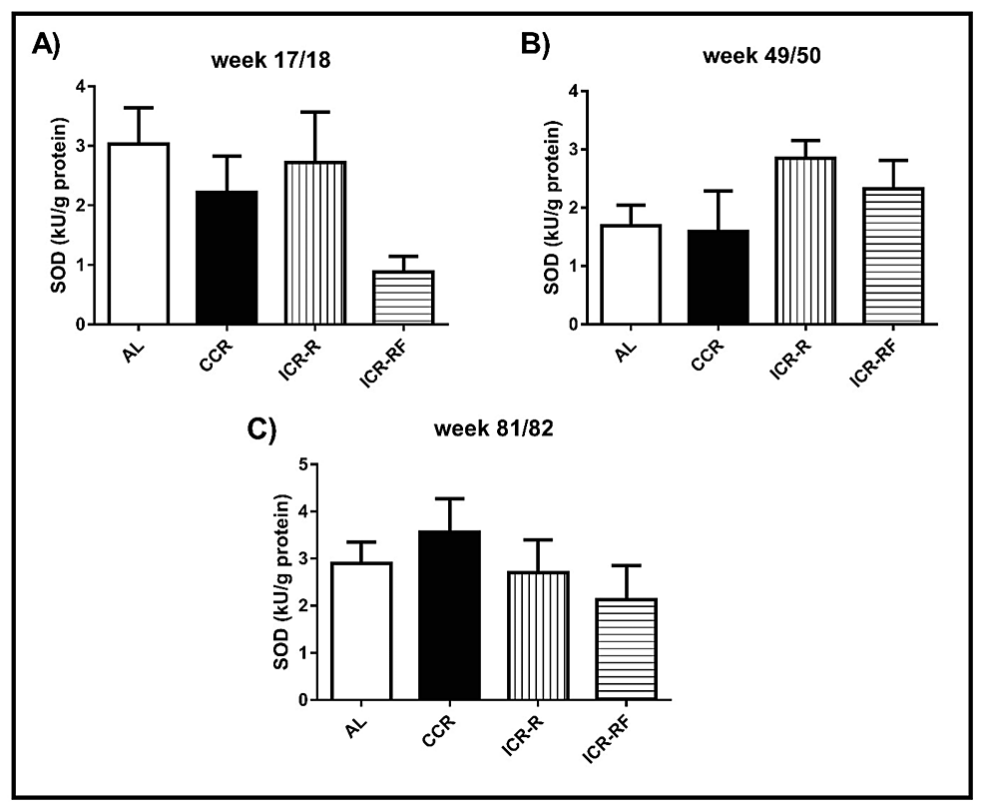
Effects of different types of calorie restriction on SOD activity in liver Effects of different CR types on SOD activity at week 17/18 (n = 12-15, Panel A), week 49/50 (n = 10-14, Panel B), and at week 81/82 (n = 4-11, Panel C). Values represent the mean ± SEM. One-way ANOVA followed by the Bonferroni multiple comparison test was used to perform comparisons among different groups. * represents significant difference (p < 0.05). AL = ad libitum; CCR = chronic calorie restriction; ICR-R = intermittent calorie restriction-restricted; ICR-RF = intermittent calorie restriction-refeed; SOD = superoxide dismutase; SEM = standard error of the mean; ANOVA = analysis of variance.

SOD activity levels in the AL, CCR, ICR-R, and ICR-RF groups at weeks 17/18, 49/50, and 81/82 were examined (Figure 3). Different CR protocols did not significantly affect the liver SOD activity at weeks 17/18 (Figure 3A), 49/50 (Figure 3B), and 81/82 (Figure 3C) (p > 0.05). However, SOD activity levels were 3.4-, 2.5-, and 3.1-fold higher in AL, CCR, and ICR-R groups, respectively, relative to the ICR-RF group at 17/18 weeks (p > 0.05, Figure 3A).

GSH levels in liver

**Figure 4:**
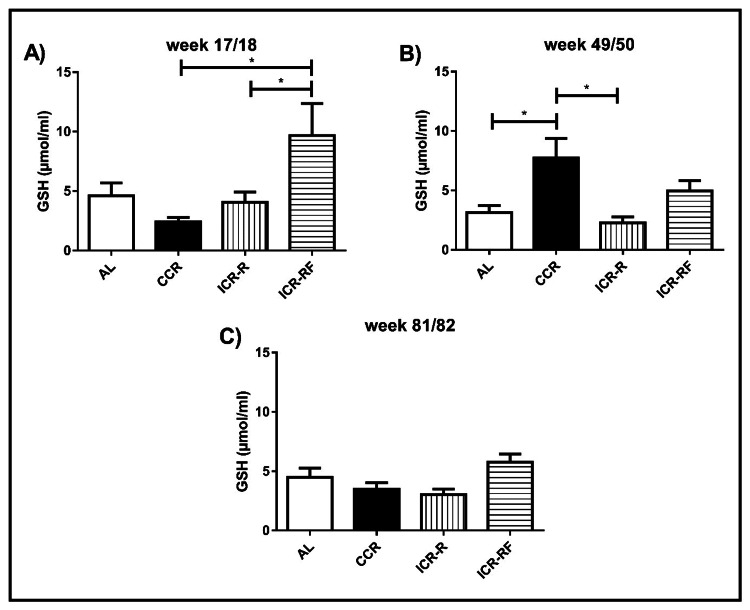
Effects of different types of calorie restriction on GSH level in liver Effects of different CR types on GSH level at week 17/18 (n = 12-14, Panel A), week 49/50 (n = 10-14, Panel B), and week 81/82 (n = 4-11, Panel C). Values represent the mean ± SEM. One-way ANOVA followed by the Bonferroni multiple comparison test was used to perform comparisons among different groups. * represents significant difference (p = 0.0059). AL = ad libitum; CCR = chronic calorie restriction; ICR-R = intermittent calorie restriction-restricted; ICR-RF = intermittent calorie restriction-referred; GSH = glutathione; SEM = standard error of the mean; ANOVA = analysis of variance.

Liver GSH levels were measured in all dietary groups at weeks 17/18, 49/50, and 81/82 (Figure 4). GSH level in the ICR-RF group was significantly higher compared to the CCR and ICR-R groups (by 75% and 58%, respectively) at 17/18 weeks of age (p < 0.05, Figure 4A). CCR group had significantly higher liver GSH levels compared to AL and ICR-R groups (by 60% and 71%, respectively) at 49/50 weeks of age (p < 0.05, Figure 4B). Neither of the CR protocols had any significant effect on liver GSH levels at 81/82 weeks (Figure 4C).

GSH-Px activity levels in liver

**Figure 5:**
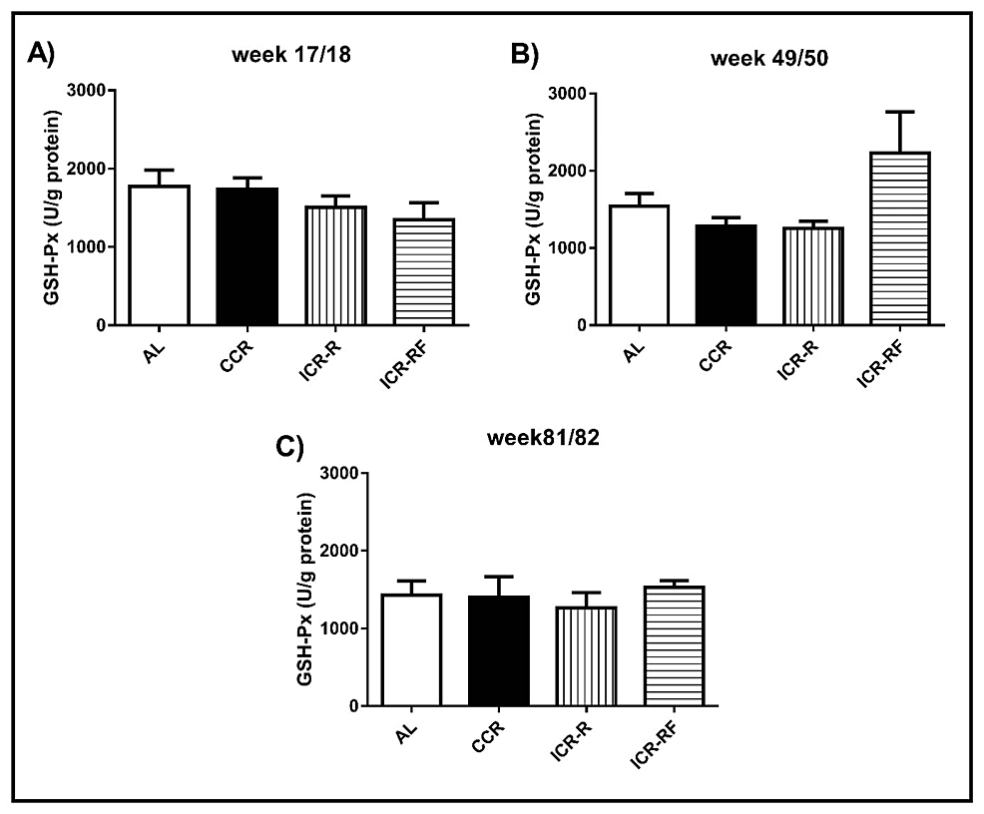
Effects of different types of calorie restriction on GSH-Px activity in liver Effects of different types of CR on GSH-Px activity at weeks 17/18 (n = 12-14, Panel A), 49/50 (n = 10-14, Panel B), and 81/82 (n = 4-11, Panel C). Values represent the mean ± SEM. One-way ANOVA followed by the Bonferroni multiple comparison test was used to perform comparisons among different groups. * represents significant difference (p < 0.05). AL = ad libitum; CCR = chronic calorie restriction; ICR-R = intermittent calorie restriction-restricted; ICR-RF = intermittent calorie restriction-refeed; GSH-Px = glutathione peroxidase; SEM = standard error of the mean; ANOVA = analysis of variance.

Differences in liver GSH-Px activity were not statistically significant among the AL, CCR, ICR-R, and ICR-RF groups at 17/18 (Figure 5A), 49/50 (Figure 5B), and 81/82 (Figure 5C) weeks of age (p > 0.05).

Effects of aging on oxidative stress parameters

The liver MDA levels were not significantly changed by aging either in AL or CCR groups (p > 0.05, Table 1). On the other hand, the MDA level was affected by aging in ICR groups (ICR-R and ICR-RF) in which the MDA level was significantly increased by about 80-90% at 49/50 weeks of age, then decreased again at week 81/82 (p < 0.05, Table 1). Although liver CAT activity was decreased by approximately twofold at 81/82 weeks of age compared to week 10 in all dietary groups, this decrease was significant only in the CCR group (p < 0.05, Table 1).

**Table 1:** Effects of aging on oxidative stress parameters in mice subjected to different types of calorie restriction Effects of aging on oxidative stress parameters in mice subjected to different types of calorie restriction. ^a,b,ab ^= Different letters within the same row represent significant differences among the groups within the same row  (p = 0.026 and p = 0.02 for MDA for ICR-R and ICR-RF groups, respectively; p = 0.007 for CAT for CCR group; p = 0.001, p = 0.003, p = 0.002, p = 0.0002 for SOD for AL, CCR, ICR-R, and ICR-RF groups, respectively; p = 0.008 for GSH for CCR group). Values represent the mean ± SEM. One-way ANOVA followed by the Bonferroni multiple comparison test was used to perform comparisons among different groups. AL = ad libitum; CCR = chronic calorie restriction; ICR-R = intermittent calorie restriction-restricted; ICR-RF = intermittent calorie restriction-refeed; MDA = malondialdehyde; CAT = catalase; SOD = superoxide dismutase; GSH = glutathione; GSH-Px = glutathione peroxidase; SEM = standard error of the mean; ANOVA = analysis of variance.

Oxidative stress parameter	Dietary group	Week			
		10	17/18	49/50	81/82
MDA	AL	43.3 ± 10.9^a^	51.2 ± 8.5	51.4 ± 7.2	47.1 ± 7.3
	CCR		52.9 ± 5.1	54.2 ± 5.4	51.3 ± 6.6
	ICR-R		42.3 ± 6.3^a^	81.4 ± 11.9^b^	40.6 ± 6.4^a^
	ICR-RF		30.2 ± 4.8^a^	72.9 ± 7.4^b^	64.3 ± 14.4^ab^
CAT	AL	517.9 ± 105.4^a^	282.6 ± 56.6	341.2 ± 51.8	294.7 ± 44.9
	CCR		266.0 ± 28.8^b^	292.3 ± 56.5^ab^	216.1 ± 36.3^b^
	ICR-R		305.4 ± 31	333.7 ± 53.2	240.4 ± 40.9
	ICR-RF		310.2 ± 47.5	297.3 ± 55.6	282.2 ± 34.6
SOD	AL	5.8 ± 0.9^a^	3.0 ± 0.6^b^	1.6 ± 0.3^b^	2.9 ± 0.4^b^
	CCR		2.2 ± 0.6^b^	1.6 ± 0.7^b^	3.6 ± 0.7^ab^
	ICR-R		2.7 ± 0.8^b^	2.9 ± 0.3^b^	2.7 ± 0.7^b^
	ICR-RF		0.9 ± 0.3^b^	2.3 ± 0.5^b^	2.1 ± 0.7^b^
GSH	AL	5.4 ± 1.3^ab^	4.6 ± 1.1	3.1 ± 0.6	4.5 ± 0.8
	CCR		2.4 ± 0.4^a^	7.7 ± 1.7^b^	3.4 ± 0.6^a^
	ICR-R		4.1 ± 0.9	2.3 ± 0.6	3.0 ± 0.5
	ICR-RF		9.7 ± 2.7	5.0 ± 0.9	5.8 ± 0.7
GSH-Px	AL	1693 ± 173.6	1776 ± 208	1545 ± 164	1426 ± 183
	CCR		1737 ± 147	1287 ± 107	1403 ± 262
	ICR-R		1507 ± 144	1251 ± 95	1266 ± 196
	ICR-RF		1346 ± 220	2229 ± 534	1527 ± 90

Liver SOD activity was significantly reduced by aging in all dietary groups: In the AL group, SOD activity was decreased by 48%, 71%, and 51% in 17/18, 49/50, and 81/82 weeks of age, respectively, compared to 10 weeks of age (baseline) (p < 0.05, Table 1). In the CCR group, SOD activity in the liver was decreased by 62% and 73% only at weeks 17/18 and 49/50, respectively, compared to baseline (p < 0.05). Similar to AL and CCR groups, SOD activity was also decreased by aging by 54%, 52%, and 54% at 17/18, 49/50, and 81/82 weeks of age, respectively, compared to baseline in the ICR-R group (p < 0.05). In addition, SOD activity in the ICR-RF group was decreased by aging by 85%, 61%, and 64% at 17/18, 49/50, and 81/82 weeks of age, respectively, compared to baseline (p < 0.05, Table 1). On the other hand, liver aging did not have any significant effects on GSH levels neither in AL nor in ICR-R groups (p > 0.05). However, GSH levels in the CCR group were increased significantly by aging until weeks 49/50 and then decreased again at week 81/82 (p < 0.05, Table 1). Although not statistically significant, liver GSH level in the ICR-RF group increased approximately twofold at 17/18 weeks of age compared to baseline (p > 0.05, Table 1), then decreased at 49/50 weeks and stay at a similar level until 81/82 weeks. Liver GHS-Px activity was not affected significantly by aging in any of the dietary groups (p > 0.05, Table 1).

Correlation between oxidative stress parameters

Correlation analyses performed between two individual oxidative stress parameters in liver samples revealed significant positive correlations between MDA and CAT (r = 0.413, p = 0.002), MDA and GSH (r = 0.338, p = 0.019), SOD and CAT (r = 0.35, p = 0.046), and CAT and GSH (r = 0454, p = 0.002) at 17/18 weeks of age (Table 2). No significant correlation was found between any oxidative stress parameters at 49/50 weeks of age. However, there was a significant positive correlation between MDA and GSH-Px at week 81/82 (r = 0.397, p = 0.024) (Table 2).

**Table 2:** Correlation between oxidative stress parameters in liver Correlation values between MDA vs SOD, MDA vs CAT, MDA vs GSH, SOD vs CAT, SOD vs GSH, and CAT vs GSH at 17/18, 49/50, and 81/82 weeks of age. All data from different CR groups were pooled together for each time point. The correlation between two parameters was analyzed using the Pearson's correlation test and linear regression analysis separately. Differences are considered statistically significant when p <0.05. MDA = malondialdehyde; CAT = catalase; SOD = superoxide dismutase; GSH = glutathione; GSH-Px = glutathione peroxidase.

Oxidative stress parameters	Week 17/18		Week 49/50		Week 81/82	
	r	p-Value	r	p-Value	r	p-Value
MDA vs SOD	-0.103	0.545	0.076	0.667	0.172	0.310
MDA vs CAT	0.413	0.002*	0.246	0.143	0.078	0.656
MDA vs GSH	0.338	0.019*	0.004	0.978	-0.037	0.826
SOD vs CAT	0.350	0.046	0.300	0.120	-0.061	0.746
SOD vs GSH	-0.114	0.540	0.120	0.522	-0.0008	0.997
CAT vs GSH	0.454	0.002	-0.070	0.690	0.311	0.079
MDA vs GSH-Px	0.256	0.089	-0.060	0.790	0.397	0.024
SOD vs GSH-Px	0.013	0.939	-0.237	0.300	0.224	0.242
CAT vs GSH-Px	-0.013	0.932	-0.182	0.470	0.217	0.320
GSH vs GSH-Px	-0.170	0.320	-0.0003	0.998	-0.139	0.518

## Discussion

CR is one of the most effective interventions for extending lifespan and delaying age-related diseases including cancer. CR is known to alter several processes such as inflammation, cell proliferation, DNA repair mechanism, and apoptosis [19]. On the other hand, CR has been reported to decrease ROS generation and enhance the antioxidant defense capacity, resulting in reduced oxidative stress [20]. Most studies apply CCR, which is a daily, consistent reduction in caloric intake. The effects of ICR - which consists of alternating periods of reduced caloric intake followed by ad libitum feeding - on oxidative stress are less well studied and results vary among different studies. It is crucial to elucidate the underlying molecular mechanism(s) of the preventive effects of CR as well as the manner in which the CR is implemented to develop better strategies to prevent and treat a number of diseases, including cancer, and promote healthy aging. The present study aims to examine the impact of CCR and ICR as well as aging on the parameters of oxidative stress in the liver of a transgenic mouse model for breast cancer, MMTV-TGF-α, in order to study the systemic effects of CR.

MDA, a commonly used biomarker of lipid peroxidation, is a secondary by-product of lipid peroxidation, which occurs as a result of radical attack [21]. We showed that liver MDA levels were not significantly affected by the application of different CR types. Previous studies have also reported findings similar to our current data. For example, the application of 25% and 50% CR for 14 days was reported to have no significant effect on the liver MDA levels in rats [22]. Another study by Stankovic et al. reported that neither 10-20% nor 30-40% CR had any significant effect on liver MDA levels compared to AL-fed male rats [23]. On the other hand, increased MDA level was reported in male rats following 50-60% CR [23]. There are also studies reporting reduced MDA levels due to CR. There are only a limited number of studies investigating the effects of intermittent fasting (IF) on oxidative stress and none of the IF protocols used in those studies are similar to the ICR protocol applied in the current study. In this context, Chausse et al. showed that alternate-day fasting for four weeks reduced MDA levels in heart tissue, but no significant change was determined in the brain, muscle, or liver tissues [24]. However, hippocampal MDA levels were reported to be reduced upon one-week application of alternate-day fasting in rats [25].

CAT is an important enzyme in the antioxidant system. No significant effects of different CR types on liver CAT activity were determined in the present study. However, aging led to decreased CAT activity in all CR groups. In line with our data, Schloesser et al. showed that 30% CR for six months did not significantly affect the liver CAT activity in C57BL6/J mice [26]. However, there are also studies reporting significant effects of CR on CAT activity. For instance, Doguc et al. showed increased erythrocyte CAT activity in mice following 60% CR for 10 weeks [27], whereas Mitchell et al. reported reduced CAT activity in the liver of 40% of CR mice compared to AL-fed mice [28]. Additionally, data from Chausse et al. indicated that CAT activity was decreased in the brain tissue due to alternate-day fasting, while IF protocol application did not have any significant effect on the activity of CAT in the heart, muscle, and liver tissues of mice [24].

SOD is a major enzymatic antioxidant, which catalyzes the dismutation of superoxide radicals into oxygen and hydrogen peroxide, providing cellular defense against ROS. In the present study, different types of CR did not have any significant effect on the SOD activity in the liver. However, aging reduced SOD activity in all dietary groups. Similarly, the application of 40% CR for two weeks was reported to have no significant effect on SOD activity in mouse heart tissue [29]. Stankovic et al. examined the effects of CR at different levels for five weeks on the SOD activity in rat liver and reported reduced SOD activity by 50-60% CR, while 10-20% CR did not have any significant effect on SOD activity [23]. Moreover, in a study conducted by Mitchell et al., liver SOD activity in mice was found to be decreased following the three-month application of either 20% or 40% CR [28]. There are also studies suggesting an enhancing effect of CR on SOD activity. For instance, Zanetti et al. reported increased SOD activity following 26% CR for three weeks compared to AL feeding in 24-month-old rats. They also showed that SOD activity in rat aorta was decreased with age, as 24-month-old rats had lower SOD activity compared to the six-month-old rats [30], which was similar to our current findings. However, contrary to our results, Descamps et al. reported that IF for four months significantly increased SOD activity in spleen mitochondria by 27% compared to AL feeding, while SOD activity in the liver was decreased by 29% in the IF group compared to AL [31].

GSH, a tripeptide, is one of the most vital antioxidants produced in the body. Our data revealed that liver GSH levels were not significantly affected by aging except that the level of GSH in the liver was higher in the CCR group at 49/50 weeks relative to weeks 17/18 and 81/82. Previous studies investigating the influence of CR on GSH levels in different tissues revealed conflicting results: Doguc et al. reported that a 10-week application of 60% CR did not significantly affect the erythrocyte GSH levels in rats [27]. On the other hand, Stankovic et al. showed that a five-week application of 30-40% CR led to increased levels of liver GSH in rats compared to the AL-fed group, whereas a five-week application of either 10-20% or 50-60% CR did not significantly affect the liver GSH levels [23]. The effects of IF on GSH levels in different tissues were also investigated previously. Data from Hu et al. suggested that IF application led to an increase in hippocampal GSH levels in 18-week-old male rats [25]. Chausse et al. reported that GSH level was increased only in liver tissue upon alternate-day fasting for one month, while no significant effect was detected in brain, muscle, and heart tissues of eight-week-old male rats [24]. In contrast to these two studies, our data suggest that ICR does not have any significant effect on liver GSH levels.

GSH-Px is an enzymatic antioxidant with a free radical scavenging capacity. We showed that liver GSH-Px activity was not significantly affected by different types of CR. Likewise, the GSH-Px activity in the liver was not significantly affected by aging in any dietary group. Similar to our results, Descamps et al. reported that four months of IF did not have any significant effects on the liver GSH-Px activity in mice [31]. On contrary, another study by Guo et al. reported that 40% CR decreased aortic GSH-Px activity in 26-month-old mice relative to AL-fed mice of the same age. They also showed that aging increased the activity of GSH-Px in AL-fed mice [32]. Data from Chausse et al. showed that the GSH-Px activities in heart, brain, and liver tissues of eight-week-old male rats were not significantly affected by one-month IF application [24]. These results suggest that CR effects on GSH-Px could be tissue-specific rather than systemic.

Our data suggest that age-dependent lipid peroxidation is associated with a reduction in antioxidant activity, especially in the ICR-applied group of mice. These data are consistent with previous studies reporting an increase in oxidative damage and decrease in antioxidant capacity with aging, as well as the oxidative stress theory of aging, which suggests that the major process promoting cellular aging is the oxidative damage to intracellular macromolecules due to slow accumulation of ROS [9]. However, lipid peroxidation was not altered by aging in the CCR group despite the decreased antioxidant levels with aging. This might be due to the fact that antioxidants were involved in the scavenging of ROS accumulated with aging and, along with the CCR protocol applied, they were sufficient to suppress the oxidative damage. This suppression was also supported by an aging-related increase in GSH levels in the CCR group. We conclude that the CCR protocol is preventive against aging-related oxidative damage, which is linked to the development of age-related diseases, including cancer. Indeed, we observed that mice in the CCR group had a lower MT incidence rate compared to the other groups. Previous studies investigating MT development, including the ones from our group, generally use MT-free mice to compare the physiological effects of different feeding protocols. The mice used in the current study were also MT-free, although they were TGF-α positive and they mostly would have developed MT if they were kept alive longer. The decreased MT rate in the CCR group may be explained by the elevated GSH levels at weeks 49/50 in this group, as GSH depletion increases susceptibility to oxidative stress, which is involved in cancer progression [33]. Enhancement of the SOD levels in the CCR group at weeks 81/82 may also contribute to the reduced tumor incidence rate in this group, since increased SOD activity has been linked to cancer prevention, including breast cancer [34]. Finally, the correlation, either negative or positive, between the oxidative stress parameters indicates that the molecules of the oxidative signaling pathway work as a system in coordination, rather than independently. 

## Conclusions

A comparison of long-term (up to 72 weeks) effects of two different CR types (CCR and ICR) on oxidative stress and aging in a mouse model of breast cancer was reported in the current study. Previous studies investigating the effects of CR on oxidative stress have reported conflicting results, which may be due to the differences in the study design such as the amount of CR applied, the age of animals when the CR application was started, duration of the CR application, as well as the species, gender, and the tissues of the animals examined. In the present paper, we report that the type of CR applied is a crucial factor affecting the impact of CR on oxidative stress and aging in the liver tissue. Understanding the mechanisms underlying the protective effects of CR against several diseases, including cancer, in detail is vital in order to develop more effective drugs, therapies, and preventive strategies. Modulation of the local rather than systemic effects of oxidative stress may be more important for the preventive effects of CR against cancer development. Further studies are needed to better understand the underlying molecular mechanism(s) of the roles of dietary intervention in age-associated diseases.

## References

[1] Taormina G, Mirisola MG (2014). Calorie restriction in mammals and simple model organisms. Biomed Res Int.

[2] Madeo F, Carmona-Gutierrez D, Hofer SJ, Kroemer G (2019). Caloric restriction mimetics against age-associated disease: targets, mechanisms, and therapeutic potential. Cell Metab.

[3] Dogan S, Rogozina OP, Lokshin AE, Grande JP, Cleary MP (2010). Effects of chronic vs. intermittent calorie restriction on mammary tumor incidence and serum adiponectin and leptin levels in MMTV-TGF-α mice at different ages. Oncol Lett.

[4] Chen Y, Ling L, Su G, Han M, Fan X, Xun P, Xu G (2016). Effect of intermittent versus chronic calorie restriction on tumor incidence: a systematic review and meta-analysis of animal studies. Sci Rep.

[5] Cicekdal MB, Kazan BT, Tuna BG (2021). Effects of two types of energy restriction on methylation levels of adiponectin receptor 1 and leptin receptor overlapping transcript in a mouse mammary tumour virus-transforming growth factor-α breast cancer mouse model. Br J Nutr.

[6] Tuna BG, Atalay PB, Altunbek M, Kalkan BM, Dogan S (2017). Effects of chronic and intermittent calorie restriction on adropin levels in breast cancer. Nutr Cancer.

[7] Aruoma OI (1994). Nutrition and health aspects of free radicals and antioxidants. Food Chem Toxicol.

[8] Valko M, Leibfritz D, Moncol J, Cronin MT, Mazur M, Telser J (2007). Free radicals and antioxidants in normal physiological functions and human disease. Int J Biochem Cell Biol.

[9] Kong Y, Trabucco SE, Zhang H (2014). Oxidative stress, mitochondrial dysfunction and the mitochondria theory of aging. Interdiscip Top Gerontol.

[10] Yilmaz B, Sandal S, Carpenter DO (2012). PCB 9 exposure induces endothelial cell death while increasing intracellular calcium and ROS levels. Environ Toxicol.

[11] Lushchak VI (2014). Free radicals, reactive oxygen species, oxidative stress and its classification. Chem Biol Interact.

[12] Cicekdal MB, Tuna BG, Charehsaz M, Cleary MP, Aydin A, Dogan S (2019). Effects of long-term intermittent versus chronic calorie restriction on oxidative stress in a mouse cancer model. IUBMB Life.

[13] Atalay PB, Kuku G, Tuna BG (2019). Effects of carbendazim and astaxanthin co-treatment on the proliferation of MCF-7 breast cancer cells. In Vitro Cell Dev Biol Anim.

[14] Ceriello A, Motz E (2004). Is oxidative stress the pathogenic mechanism underlying insulin resistance, diabetes, and cardiovascular disease? The common soil hypothesis revisited. Arterioscler Thromb Vasc Biol.

[15] Keenan KP, Soper KA, Hertzog PR (1995). Diet, overfeeding, and moderate dietary restriction in control Sprague-Dawley rats: II. Effects on age-related proliferative and degenerative lesions. Toxicol Pathol.

[16] Dogan S, Cicekdal MB, Ozorhan U (2021). Roles of adiponectin and leptin signaling-related microRNAs in the preventive effects of calorie restriction in mammary tumor development. Appl Physiol Nutr Metab.

[17] Jamall IS, Smith JC (1985). Effects of cadmium treatment on selenium-dependent and selenium-independent glutathione peroxidase activities and lipid peroxidation in the kidney and liver of rats maintained on various levels of dietary selenium. Arch Toxicol.

[18] Sun Y, Oberley LW, Li Y (1988). A simple method for clinical assay of superoxide dismutase. Clin Chem.

[19] Bruss MD, Thompson AC, Aggarwal I, Khambatta CF, Hellerstein MK (2011). The effects of physiological adaptations to calorie restriction on global cell proliferation rates. Am J Physiol Endocrinol Metab.

[20] Gredilla R, Barja G (2005). Minireview: the role of oxidative stress in relation to caloric restriction and longevity. Endocrinology.

[21] Ayala A, Muñoz MF, Argüelles S (2014). Lipid peroxidation: production, metabolism, and signaling mechanisms of malondialdehyde and 4-hydroxy-2-nonenal. Oxid Med Cell Longev.

[22] Ling PR, Bistrian BR (2009). Comparison of the effects of food versus protein restriction on selected nutritional and inflammatory markers in rats. Metabolism.

[23] Stankovic M, Mladenovic D, Ninkovic M, Vucevic D, Tomasevic T, Radosavljevic T (2013). Effects of caloric restriction on oxidative stress parameters. Gen Physiol Biophys.

[24] Chausse B, Vieira-Lara MA, Sanchez AB, Medeiros MH, Kowaltowski AJ (2015). Intermittent fasting results in tissue-specific changes in bioenergetics and redox state. PLoS One.

[25] Hu Y, Zhang M, Chen Y, Yang Y, Zhang JJ (2019). Postoperative intermittent fasting prevents hippocampal oxidative stress and memory deficits in a rat model of chronic cerebral hypoperfusion. Eur J Nutr.

[26] Schloesser A, Campbell G, Glüer CC, Rimbach G, Huebbe P (2015). Restriction on an energy-dense diet improves markers of metabolic health and cellular aging in mice through decreasing hepatic mTOR activity. Rejuvenation Res.

[27] Doguc DK, Yilmaz N, Vural H, Kara Y (2013). The effect of calorie restriction on lipid peroxidation and antioxidant enzymes in rats. Sakarya Med J.

[28] Mitchell SE, Delville C, Konstantopedos P (2015). The effects of graded levels of calorie restriction: II. Impact of short term calorie and protein restriction on circulating hormone levels, glucose homeostasis and oxidative stress in male C57BL/6 mice. Oncotarget.

[29] Kobara M, Furumori-Yukiya A, Kitamura M, Matsumura M, Ohigashi M, Toba H, Nakata T (2015). Short-term caloric restriction suppresses cardiac oxidative stress and hypertrophy caused by chronic pressure overload. J Card Fail.

[30] Zanetti M, Gortan Cappellari G, Burekovic I, Barazzoni R, Stebel M, Guarnieri G (2010). Caloric restriction improves endothelial dysfunction during vascular aging: effects on nitric oxide synthase isoforms and oxidative stress in rat aorta. Exp Gerontol.

[31] Descamps O, Riondel J, Ducros V, Roussel AM (2005). Mitochondrial production of reactive oxygen species and incidence of age-associated lymphoma in OF1 mice: effect of alternate-day fasting. Mech Ageing Dev.

[32] Guo ZM, Yang H, Hamilton ML, VanRemmen H, Richardson A (2001). Effects of age and food restriction on oxidative DNA damage and antioxidant enzyme activities in the mouse aorta. Mech Ageing Dev.

[33] Sohal RS, Agarwal S, Candas M, Forster MJ, Lal H (1994). Effect of age and caloric restriction on DNA oxidative damage in different tissues of C57BL/6 mice. Mech Ageing Dev.

[34] Robbins D, Zhao Y (2014). Manganese superoxide dismutase in cancer prevention. Antioxid Redox Signal.

